# Large B-cell Lymphoma of the Jejunum: Computed tomography delivers considerable diagnostic value by showing the following typical signs of small bowel lymphoma: segmental wall thickening, aneurysmal dilatation, and locoregional lymphadenopathy

**DOI:** 10.5334/jbsr.1826

**Published:** 2019-06-28

**Authors:** Nico Hustings, Frederik Feyaerts

**Affiliations:** 1 AZ Sint-Lucas, BE

**Keywords:** Primary Gastrointestinal Lymphoma, Diffuse Large B-Cell Lymphoma, Emergency Presentation of Cancer, General Surgery, Histopathology

## Case

**Figure 1:**
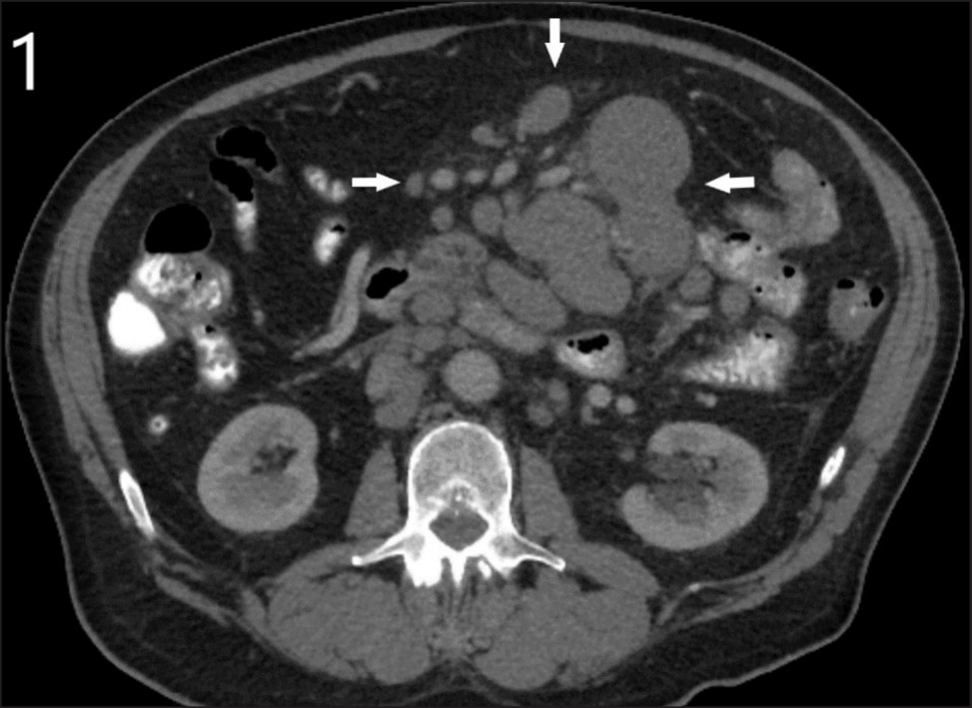


**Figure 2:**
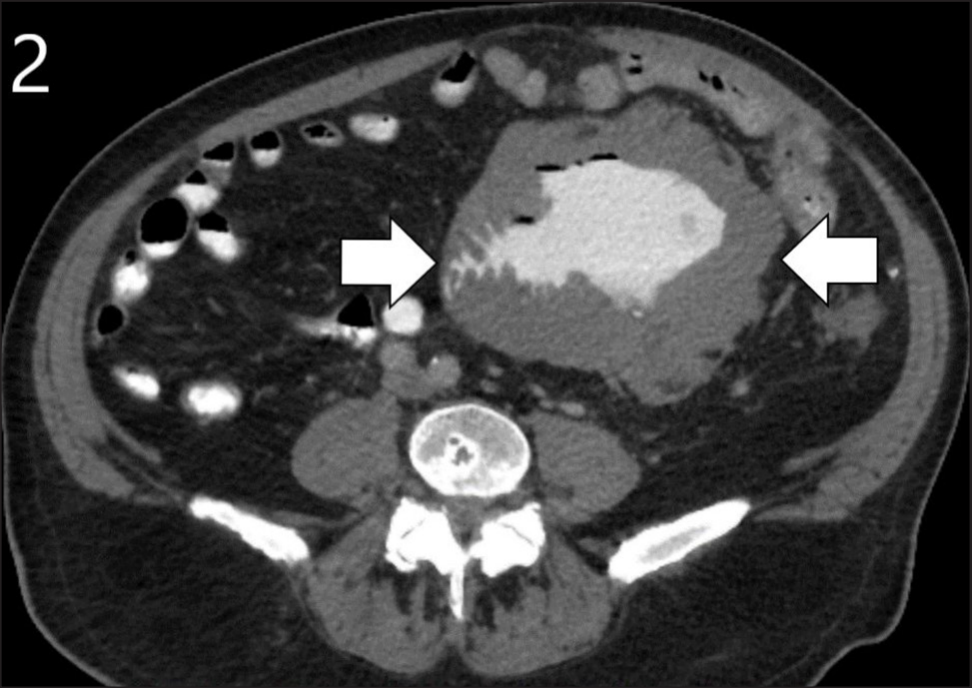


**Figure 3:**
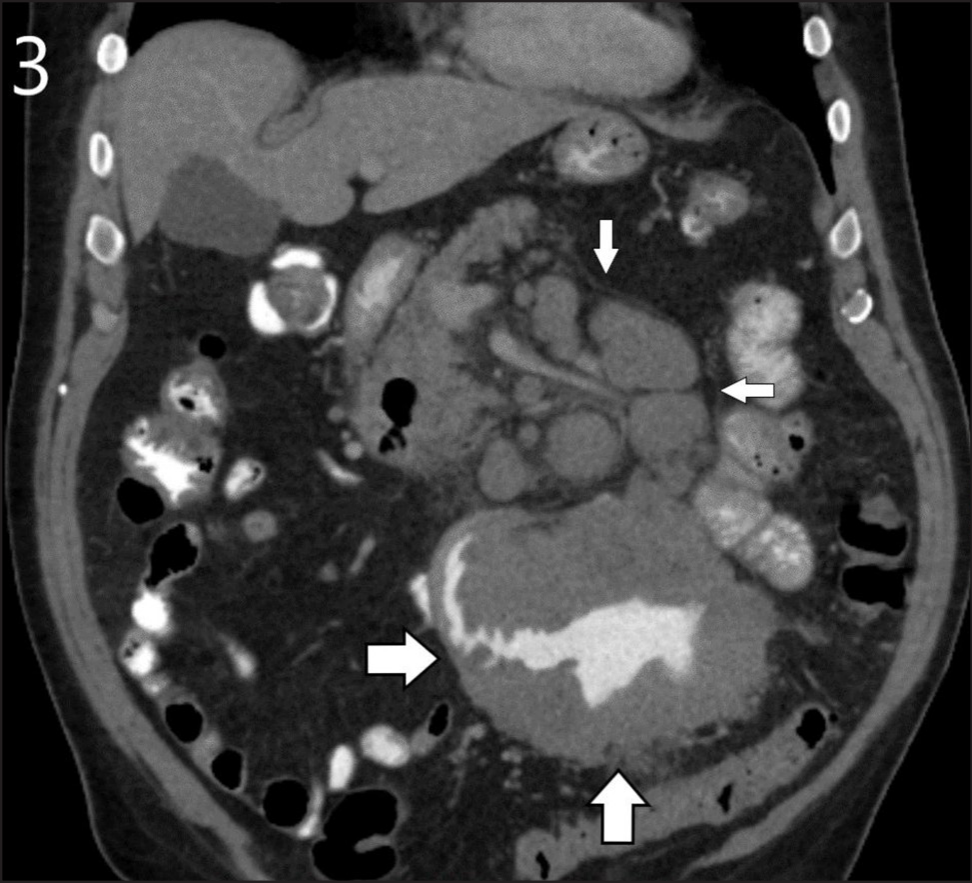


A 72-year-old male presented to the emergency department with complaints of abdominal pain for six days, decreased appetite and diarrhoea for 3–4 months. Clinical examination revealed a periumbilical mass, not yet noticed by the patient. Routine haematological tests showed a slightly elevated C-reactive protein (36 mg/L) and mild hypernatremia (149 mmol/L). The medical history mentioned a positive immunochemical faecal occult blood test (iFOBT) eight years ago that led to resection of two mildly dysplastic colon polyps. Abdominal computed tomography (CT) was performed after administration of oral and intravenous iodine-based contrast. A notable irregular bowel wall thickening was detected in a jejunal loop, extending through the entire circumference and over a length of nearly 15 cm. The wall broadening went along with widening of the lumen, making it apparent as an aneurysmal dilated tumoral mass containing air-fluid level (Figures 2 and 3, large arrows). CT also demonstrated numerous enlarged mesenteric and retroperitoneal lymph nodes (Figures 1 and 3, small arrows). A close spatial relationship was noted between the jejunal tumoral changes and the voluminous lymph nodes. These CT signs were highly suggestive of a small bowel lymphoma with locoregional lymphadenopathy. A diagnostic laparoscopy yielded similar abnormalities, and biopsies were obtained. Finally, histopathologic analysis confirmed a diffuse large B-cell lymphoma of the jejunum.

## Comment

Although small bowel tumours only represent 5% of all neoplasms in the gastrointestinal tract, lymphomas cover 25% of the primary small bowel tumours [1]. The distal ileum is most frequently affected, in line with its highest concentration of lymphoid tissue. The most important risk factors are infections such as human immunodeficiency virus, Helicobacter pylori, Epstein-Barr virus, and inflammatory conditions such as celiac disease. Small bowel lymphoma has a varied and rather unspecific presentation. The common symptoms are abdominal pain, weight loss, nausea, diarrhoea, constipation, and abdominal mass. Most cases are diagnosed late on account of the vague complaints. Acute presentation as a result of intestinal perforation or obstruction is possible, but this occurs rarely.

Abdominal CT typically shows an aneurysmal dilated segment of small bowel with irregular wall thickening. This aneurysmal dilatation is explained by the destruction of the muscularis propria or the autonomous myenteric plexus; this pathophysiologic process also explains why obstruction is uncommon. Bulky mesenteric and/or retroperitoneal lymphadenopathy is often demonstrated in small bowel lymphoma, in approximately 50% of the cases. Atypical small bowel lymphomas present as solid polypoid or eccentric masses, making it mandatory to differentiate these lesions from a small intestine adenocarcinoma. Extensive lymphadenopathy and splenomegaly indicate lymphoma, while contiguous mesenteric fat infiltration is suggestive of adenocarcinoma. The definitive diagnosis is based on the outcome of histopathological analysis. The treatment primarily consists of surgical excision or chemoradiotherapy.
